# Elements of the cellular metabolic structure

**DOI:** 10.3389/fmolb.2015.00016

**Published:** 2015-04-28

**Authors:** Ildefonso M. De la Fuente

**Affiliations:** ^1^Department of Cell Biology and Immunology, Institute of Parasitology and Biomedicine “López-Neyra,” Consejo Superior de Investigaciones CientíficasGranada, Spain; ^2^Department of Mathematics, University of the Basque Country, UPV/Euskal Herriko UnibertsitateaLeioa, Spain

**Keywords:** systems biology, metabolic networks, systemic metabolism, Hopfield dynamics, dissipative processes, self-organization

## Abstract

A large number of studies have demonstrated the existence of metabolic covalent modifications in different molecular structures, which are able to store biochemical information that is not encoded by DNA. Some of these covalent mark patterns can be transmitted across generations (epigenetic changes). Recently, the emergence of Hopfield-like attractor dynamics has been observed in self-organized enzymatic networks, which have the capacity to store functional catalytic patterns that can be correctly recovered by specific input stimuli. Hopfield-like metabolic dynamics are stable and can be maintained as a long-term biochemical memory. In addition, specific molecular information can be transferred from the functional dynamics of the metabolic networks to the enzymatic activity involved in covalent post-translational modulation, so that determined functional memory can be embedded in multiple stable molecular marks. The metabolic dynamics governed by Hopfield-type attractors (functional processes), as well as the enzymatic covalent modifications of specific molecules (structural dynamic processes) seem to represent the two stages of the dynamical memory of cellular metabolism (metabolic memory). Epigenetic processes appear to be the structural manifestation of this cellular metabolic memory. Here, a new framework for molecular information storage in the cell is presented, which is characterized by two functionally and molecularly interrelated systems: a dynamic, flexible and adaptive system (metabolic memory) and an essentially conservative system (genetic memory). The molecular information of both systems seems to coordinate the physiological development of the whole cell.

## Enzymatic self-organization

### Multienzymatic complexes

Living cells are essentially highly evolved dynamic metabolic reactors, in which most of the biomolecules are synthesized and destroyed by means of interrelated enzymatic processes, which are densely integrated in modular networks that shape one of the most complex dynamic systems in nature (De la Fuente, 2014).

From a functional point of view, enzymes are the main molecules of this amazing biochemical reactor. They are responsible for almost all biomolecular transformations, which, when globally considered, are called cellular metabolism. Most enzymes are proteins, but a few RNA molecules called ribozymes, also manifest catalytic activity. How the enzymes are organized under the complex conditions prevailing inside the cell is a crucial issue for understanding the fundamental functional architecture of cellular life.

For many years, it has been assumed that enzymes work in an isolated way. However, intensive studies of protein-protein interactions have shown that the internal cellular medium is an assembly of supra-molecular protein complexes (Gavin and Superti-Furga, 2003; Segura et al., 2010; Völkel et al., 2010; Ramisetty and Washburn, 2011), where homologous or heterologous protein-protein interactions are the rule (Pang et al., 2008). Thus, analyses of the proteome of *Saccharomyces cerevisiae* have shown that at least 83% of proteins form complexes comprised of 2–83 proteins (Gavin et al., 2002). This kind of associative organization occurs in all sorts of cells, both eukaryotes and prokaryotes (Uetz et al., 2000; Ito et al., 2001; Ho et al., 2002; Bobik, 2006; Sutter et al., 2008; Yeates et al., 2008).

Complexes formed by multienzymatic associations may allow for substrate channeling, i.e., direct transfer of intermediate metabolites from the active site of one enzyme to the next without prior diffusion into the bulk medium. Metabolic channeling decreases the transit time of reaction substrates, thus increasing the speed and efficiency of catalysis by avoiding delays due to the diffusion of reaction intermediates and their eventual loss (Clegg and Jackson, 1990; Ovadi and Srere, 2000; Jorgensen et al., 2005; Jovanović et al., 2007). Substrate channeling may also occur within channels or on the electrostatic surface of enzyme complexes (Milani et al., 2003; Ishikawa et al., 2004; Beg et al., 2007).

Additionally, reversible interactions between multienzyme complexes and structural proteins or membranes occur frequently in eukaryotic cells leading to the emergence of metabolic microcompartments (Verkman, 2002; Lunn, 2007; Monge et al., 2008, 2009; Saks et al., 2009). For instance, the nuclear processes such as DNA replication, DNA repair and transcription of RNAs are organized in dynamic microenvironments that are functionally connected to the ubiquitin-proteasome system (Zhao et al., 2009). Microcompartmentation also occurs in prokaryotes, but in this case it consists of protein “shells” composed of thousands of protein subunits (Yeates et al., 2007; Fan et al., 2010). The dynamics of molecular processes that intervene in microcompartmentation and its maintenance remain unclear.

The concept of multienzymatic associations was first conceived in 1970 by Kuzin (1970) and adopted in 1972 by Srere (1972, 1985) who suggested the use of the term metabolon to refer to an organized multienzyme complex belonging to a metabolic sequence. A wide variety of studies have revealed the importance of multienzymatic associations in almost all cellular processes (Veliky et al., 2007; Pflieger et al., 2011). Various examples of such associations are detailed in Table 1.

**Table 1:** **Examples of multienzymatic associations in published studies**.

**Multienzymatic association**	**Study**
Purinosome	Zhao et al., 2013
Aminoacyl-tRNAsynthetase complex	Bhaskaran and Perona, 2011
Urea cycle	Cheung et al., 1989
ATP synthasome	Clémençon, 2012
Respiratory chain supercomplex	Schägger, 2002; Stuart, 2008; Bultema et al., 2009
Benson-Calvin cycle	Suss et al., 1993; Graciet et al., 2004
Replisome	Murthy and Pasupathy, 1994-1995
Amino acid catabolism complex	Islam et al., 2007
Cellulosome	Bayer et al., 2004
Sporopollenin biosynthesis complex	Lallemand et al., 2013
Spliceosome	Will and Lührmann, 2011
21 S complex of enzymes for DNA synthesis	Li et al., 1993
TRAMP complex	Jia et al., 2012
Glycogen biosynthesis complex	Wilson et al., 2010
Degradosome	Carpousis, 2002
Vault ribonucleoprotein complex	Zheng et al., 2005
Proteasome	Murata et al., 2009
Fatty acid oxidation complex	Binstock and Schulz, 1981; Parker and Engel, 2000
Ribosome	Lin et al., 2013
Glycolytic enzymes associate	Waingeh et al., 2006; Graham et al., 2007; Forlemu et al., 2011
Protein kinase complexes	Rohila et al., 2009
Photosystem I	Fromme and Mathis, 2004
Krebs cycle	Barnes and Weitzman, 1986; Beeckmans et al., 1990; Mitchell, 1996
COP9 signalosome complex	Schwechheimer, 2004
Ski complex	Halbach et al., 2013
Exosome	Makino et al., 2013
Dystrophin-associated protein complex	Ehmsen et al., 2002
Arginine biosynthesis complex	Abadjieva et al., 2001
Mitotic and cytokinetic protein complexes	D'Avino et al., 2009

Recently, analysis of conserved sequences in biochemical reactions has shown that cellular metabolism contains basic functional units which tend to correspond to traditional metabolic pathways. These functional multi-enzymatic units seem to be the catalytic building blocks of cellular metabolism (Kanehisa, 2013; Muto et al., 2013). Another kind of functional catalytic association is the interactome which refers to protein–protein interaction networks (unlike metabolic pathways, that represent a sequence of molecular interactions leading to a final product) (Van Leene et al., 2007; Bonetta, 2010; Gautam et al., 2012; Sharma et al., 2013).

### Temporal self-organization of multienzymatic processes

Enzymatic organization at the molecular level presents another relevant characteristic: the emergence of dissipative catalytic structures. Experimental observations have shown that enzymes shape functional catalytic associations in which molecular rhythms and quasi-steady states may spontaneously emerge far from thermodynamic equilibrium. In fact, under cellular conditions, the temporal evolution of practically all metabolite concentrations exhibits marked variations, presenting transitions between quasi-steady states and oscillatory behaviors (De la Fuente, 2014). In the quasi-stationary states, the concentration of any given metabolite drifts over time in a non-oscillatory way. Not long ago, the use of nanobiosensors to provide real-time measurements of specific intracellular metabolites in intact cells, revealed complex oscillations and quasi-steady states, but importantly, these patterns were never found to be constant (Ozalp et al., 2010).

Metabolic rhythms constitute a genuine manifestation of dissipative self-organization in the enzymatic activities of the cell. In general terms, self-organization can be defined as the spontaneous emergence of macroscopic non-equilibrium dynamic structures, as a result of the collective behavior of elements interacting nonlinearly with each other, to generate a system which increases its structural and functional complexity, driven by energy dissipation (Halley and Winkler, 2008; Misteli, 2009; De la Fuente, 2014).

An important ingredient of metabolic self-organization is the nonlinear interaction between metabolites and enzymes, involving autocatalysis, feed-back processes, and allosteric regulation, among others. It is well established that non-equilibrium states can be a source of order in the sense that the irreversible processes may lead to a new type of dynamic state in which the system becomes ordered in space and time. In fact, the non-linearity associated with irreversible enzymatic reactions seems to be an essential mechanism which may allow dissipative metabolic organization far from thermodynamic equilibrium (Goldbeter, 2002, 2007).

Self-organization is based on the concept of dissipative structures, and its theoretical roots can be traced back to the Nobel Prize Laureate in Chemistry Ilya Prigogine (Nicolis and Prigogine, 1977). There exist different kinds of metabolic dissipative structures under cellular conditions, including oscillations in metabolite concentrations, molecular circadian rhythms and spatial biochemical waves. During the last four decades, extensive studies of biochemical dynamics both in prokaryotic and eukaryotic cells have revealed that most of the fundamental metabolic processes exhibit oscillations (temporal self-organizations). Various examples of molecular oscillations are detailed in Table 2.

**Table 2:** **Examples of temporal self-organization in metabolic processes**.

**Metabolic processes**	**Study**
Free fatty acids	Getty-Kaushik et al., 2005
NAD(P)H concentration	Rosenspire et al., 2001
Biosynthesis of phospholipids	Marquez et al., 2004
Cyclic AMP concentration	Holz et al., 2008
ATP	Ainscow et al., 2002
Adenine nucleotides	Zhaojun et al., 2004
Intracellular glutathione concentration	Lloyd and Murray, 2005
Actin polymerization	Rengan and Omann, 1999
ERK/MAPK metabolism	Shankaran et al., 2009
mRNA levels	Zhaojun et al., 2004
Intracellular free amino acid pools	Hans et al., 2003
Cytokinins	Hartig and Beck, 2005
Cyclins	Hungerbuehler et al., 2007
Transcription of cyclins	Shaul et al., 1996
Gene expression	Tonozuka et al., 2001; Klevecz et al., 2004; Tian et al., 2005; Chabot et al., 2007
Microtubule polymerization	Lange et al., 1988
Membrane receptor activities	Placantonakis and Welsh, 2001
Membrane potential	De Forest and Wheeler, 1999
Intracellular Ph	Sánchez-Armáss et al., 2006
Respiratory metabolism	Lloyd et al., 2002
Glycolysis	Dano et al., 1999
Intracellular calcium concentration	Ishii et al., 2006
Metabolism of carbohydrates	Jules et al., 2005
Beta-oxidation of fatty acids	Getty et al., 2000
Metabolism of mRNA	Klevecz and Murray, 2001
tRNA	Brodsky et al., 1992
Proteolysis	Kindzelskii et al., 1998
Urea cycle	Fuentes et al., 1994
Krebs cycle	Wittmann et al., 2005
Mitochondrial metabolic processes	Aon et al., 2008
Nuclear translocation of the transcription factor	Garmendia-Torres et al., 2007
Amino acid transports	Barril and Potter, 1968
Peroxidase-oxidase reactions	Møller et al., 1998
Protein kinase activities	Chiam and Rajagopal, 2007
Photosynthetic reactions	Smrcinová et al., 1998

Experimental observations of *S. cerevisiae* during continuous culture have also revealed the presence of oscillatory dynamics in most of the transcriptome and metabolome (Klevecz et al., 2004; Murray et al., 2007). In some studies, it has been observed that at least 60% of transcripts exhibit oscillations with an approximate period of 300 min (Tu et al., 2005). Moreover, other studies have shown that the entire transcriptome presents low-amplitude oscillatory behavior (Lloyd and Murray, 2006) and this phenomenon has been described as a genome-wide oscillation (Klevecz et al., 2004; Lloyd and Murray, 2005, 2006; Oliva et al., 2005; Tu et al., 2005; Murray et al., 2007).

Evidence that cells exhibit multi-oscillatory metabolic processes with fractal properties has also been reported. This dynamic behavior appears to be consistent with scale-free dynamics spanning a wide range of frequencies of at least three orders of magnitude (Aon et al., 2008).

The temporal organization of metabolic processes in terms of rhythmic phenomena spans periods ranging from milliseconds (Aon et al., 2006), to seconds (Roussel et al., 2006), minutes (Chance et al., 1973; Berridge and Galione, 1988) and hours (Brodsky, 2006). Oscillatory behavior from simple patterns to complex temporal structures (MacDonald et al., 2003), including bursting oscillations, with one large spike and a series of secondary oscillations (Dekhuijzen and Bagust, 1996), have often been observed. Multiple autonomous oscillatory behaviors emerge simultaneously in the cell, so that global metabolism can be considered to be a complex multi-oscillator super-system (Lloyd and Murray, 2005, 2006; Murray et al., 2007; Aon et al., 2008; Sasidharan et al., 2012; Amariei et al., 2014; De la Fuente et al., 2014).

Numerous mathematical studies of temporal metabolic rhythms have contributed to a better understanding of the self-organized enzymatic functionality which is apparent under cellular conditions (Boiteux et al., 1980; Tyson, 1991; De la Fuente et al., 1996, 1999; Heinrich and Schuster, 1996; Novak et al., 2007; Zhou et al., 2009; Goldbeter, 2013). Dissipative self-organization at the enzymatic level presents other kinds of emergent dynamic structures, such as circadian rhythms (Schibler and Sassone-Corsi, 2002; Cardone et al., 2005; De la Fuente, 2010) and spatial biochemical waves (Petty and Kindzelskii, 2001; Bernardinelli et al., 2004; Asano et al., 2008; De la Fuente, 2010). Metabolic rhythms constitute one of the hallmark properties of multienzymatic dynamics. The conditions required for the emergence and sustainability of these rhythms, and how they are regulated, represent a biological problem of the highest significance. However, in spite of its physiological importance, many aspects of cellular dissipative structures are still poorly understood and thus deserve further attention.

### Dissipative multienzymatic complexes: metabolic subsystems

Self-organized multienzymatic complexes can be viewed as dissipative structures in which molecular rhythms and functional integrative processes can emerge, increasing the efficiency and control of the biochemical reactions involved (De la Fuente, 2010; De la Fuente and Cortés, 2012). This concept was conceived in 1999 and was termed *metabolic subsystem* to refer to a dissipatively structured enzymatic set in which molecular oscillations and steady state patterns may emerge spontaneously (De la Fuente et al., 1999).

Thermodynamically, metabolic subsystems represent advantageous biochemical organizations, forming unique, well-defined dynamic systems, in which catalytic activity is autonomous with respect to other enzymatic associations. Therefore, each self-organized enzymatic set shapes a catalytic entity as a whole and carries out its activities relatively independently of others (De la Fuente, 2010, 2014). The presence of some regulatory processes (enzymes of both allosteric and covalent modulation) in each metabolic subsystem makes sophisticated interconnections among them possible. As a result of variable combinations in the different input conditions (substrate fluxes, allosteric signals, specific post-translational modulations, etc.), a rich variety of dynamic patterns, each corresponding to distinct catalytic activity regimes, can emerge in these metabolic subsystems (De la Fuente et al., 2013, 2014).

This huge variety of catalytic patterns is a *sine qua non* condition for complex information properties (with a capacity to store functional catalytic patterns) that can emerge in the cellular metabolic networks (see Section Metabolic Networks and Information Properties for more details).

Metabolic subsystems represent highly efficient nanomachines which can execute autonomous and discrete biological functions, underlying the structural and functional dynamic organization of the cell. Associations between metabolic subsystems can form higher level complex molecular organizations, which appear to occur for example, in intracellular energetic units (ICEU) (Saks et al., 2006) and synaptosomes (Monge et al., 2008).

In essence, metabolic subsystems are self-organized biochemical machines which seem to constitute a fundamental and elemental catalytic unit of the cellular biochemical reactor (De la Fuente, 2014).

## Functional organization of enzymes in metabolic networks

### Metabolic functionality

All physiological processes rely on metabolic functionality, which is a key element for understanding the dynamic principles of cellular life at all levels of its biochemical organization (Noble et al., 2014).

Enzymatic activity depends on different factors, mainly substrate concentration, temperature, pH, enzyme concentration and the presence of any inhibitors or activators. When an enzymatic system operates sufficiently far from equilibrium, oscillatory catalytic patterns can emerge. Such dynamic behaviors find their roots in non-linear regulatory processes, e.g., feedback loops, cooperativity and stoichiometric autocatalysis (Goldbeter, 2002). In addition, due to collective interactions under cellular conditions, substrate fluxes and regulatory signals shape complex topological structures in which metabolite concentration is continuously changing over time because of the dynamics that emerge in the catalytic networks (De la Fuente, 2014). This collective enzymatic connectivity makes the rate at which enzymatic reactions proceed highly complex.

As a consequence, the functional architecture of the cell is a result of all these different factors, and in particular, of the complex dynamic interactions between enzymatic processes integrated in biochemical networks. Thus, metabolic functionality at a systemic level prominently depends on the collective architecture (structural and functional) of the catalytic reactions, which is defined by the two essential principles that link the different modes of enzymatic connectivity, i.e., *metabolic segregation* and *metabolic integration*.

### Metabolic segregation

The separation of metabolic tasks and catalytic roles requires that the enzymes with correlated biochemical activities are grouped together. As a consequence, the entire metabolism is functionally segregated at multiple levels of specialization (e.g., signal transduction, oxidative phosphorylation, lysosomal metabolic activity, etc.), originating different kinds of specific modular structures. A metabolic module can be considered to be a discrete functional entity, which performs relatively autonomous processes with specific and coherent dynamic activities (Hartwell et al., 1999).

A wide variety of studies have shown that cellular metabolic networks are organized in a specialized modular fashion; this constitutes a defining characteristic of metabolic organization in the cell (Ravasz et al., 2002; Nurse, 2008; Ravasz, 2009; Hao et al., 2012; Kaltenbach and Stelling, 2012; Geryk and Slanina, 2013). Modular segregations have been observed in all the central physiological processes, e.g., the enzymatic organization of metabolic pathways (Muto et al., 2013), chaperone activities (Korcsmáros et al., 2007), chemotaxis (Postma et al., 2004; Shimizu et al., 2010), apoptosis (Harrington et al., 2008), the cell cycle (Boruc et al., 2010; Hsu et al., 2011), the Golgi apparatus (Nakamura et al., 2012), and kinetochore organization (Petrovic et al., 2014). Specific modular metabolic networks also participate in the transcriptional system, such as miRNA regulatory networks (Gennarino et al., 2012), transcription factor networks (Neph et al., 2012), RNA polymerase complexes (Schubert, 2014), and mRNA dynamics (Vlasova-St Louis and Bohjanen, 2011). Metabolic subsystems, i.e., self-organized multienzymatic associations, can be considered to be elemental catalytic modules in the cell (De la Fuente et al., 2008, 2010, 2014).

Metabolic networks may also contain canonical motifs, which represent recurrent and statistically significant sub-graphs or patterns (Milo et al., 2002; Kashtan and Alon, 2005; Alon, 2007; Masoudi-Nejad et al., 2012). These sub-graphs that repeat themselves in a specific network are defined by a specific pattern of interactions, which may reflect particular functional properties but, in contrast to modules, motifs do not function in isolation (Lacroix et al., 2006).

The functional role played by specialized metabolic networks is defined largely by its structural connections. However, at least three main kinds of metabolic connectivity have to be considered: *structural* connectivity, which is formed by structural molecular links such as substrate fluxes and regulatory signals, *functional* connectivity, which describes statistical dependencies (non-causal) between the activity patterns, and *effective* connectivity, which rests on the causal effects between different biochemical activities (De la Fuente et al., 2011, 2013). A number of information-based dynamic tools for the functionality and correlations between biochemical processes have been proposed. However, functional correlations (*functional connectivity*) do not imply *effective connectivity* because most synchronization measures do not distinguish between causal and non-causal interactions. In recent studies, transfer entropy (TE) has been proposed to be a rigorous, robust and self-consistent method for the causal quantification of functional information flow between nonlinear processes (*effective connectivity*).

The quantitative study of enzymatic effective connectivity can be considered at different biochemical organization levels, e.g., in a biochemical pathway (De la Fuente and Cortés, 2012) and in a metabolic network (De la Fuente et al., 2011). In particular, in order to investigate the functional structure (both functional and effective connectivity) of a large number of self-organized enzymes, the effective connectivity has been analyzed by means of transfer entropy in dissipative metabolic networks (DMNs) under different external conditions (De la Fuente et al., 2011).

Essentially, a DMN is a set of interconnected nodes, each one corresponding to a specific metabolic subsystem, which can be considered to be an elemental self-organized catalytic module (see Section Enzymatic Self-Organization for more details). The DMN nodes are interconnected through a complex topological structure (structural connectivity) of substrate fluxes and different regulatory mechanisms: activatory and inhibitory allosteric modulations, and post-translational modulations. In keeping with experimental observations, the emergent output activity of each enzymatic subsystem in the DMN's can be either oscillatory or steady state and comprises an infinite number of distinct activity regimes (De la Fuente et al., 2008, 2010; De la Fuente and Cortés, 2012).

Transfer entropy analysis of the DMN showed that in addition to the network's topological structure, characterized by the specific location of enzymatic subsystems, molecular substrate fluxes and regulatory signals, there is another functional structure of biomolecular information flow which is modular, dynamic and able to modify the catalytic activities of all the enzymatic sets (De la Fuente et al., 2011). In particular, certain self-organized enzymatic sets are spontaneously clustered forming functional metabolic sub-networks. Some modules are preserved under different external conditions, whereas others are preserved but with the fluxes inverted and yet other modules emerge only under specific external perturbations. These modules work efficiently: very concrete enzymatic subsystems are connected with others by means of effective information flow allowing high coordination and precise catalytic regulation. Dynamic changes between the functional modules correspond to metabolic switches, which allow for critical transitions in enzymatic activities. The modules coordinated by effective connectivity and the functional switches seem to be important regulatory mechanisms which are able to modify the catalytic activity of all the enzymatic sets (De la Fuente et al., 2011).

### Metabolic integration

In addition to functional segregation, cellular enzymatic processes exhibit another fundamental principle of metabolic connectivity, i.e., functional integration.

Catalytic systems organized into distinct modular networks seem to be integrated; as a consequence, systemic responses emerge from the concerted actions of specialized metabolic processes (Almaas et al., 2004, 2005; Yoon et al., 2007; Buescher et al., 2012; San Román et al., 2014). Whereas functional segregation is an expression of the relative autonomy of specialized biochemical networks, metabolic integration is a complementary principle that refers to significant deviations from modular network autonomy, so that the collective catalytic behavior generates coherent global patterns and molecular information that is simultaneously highly diversified and highly integrated (De la Fuente et al., 2011).

**Figure 1:**
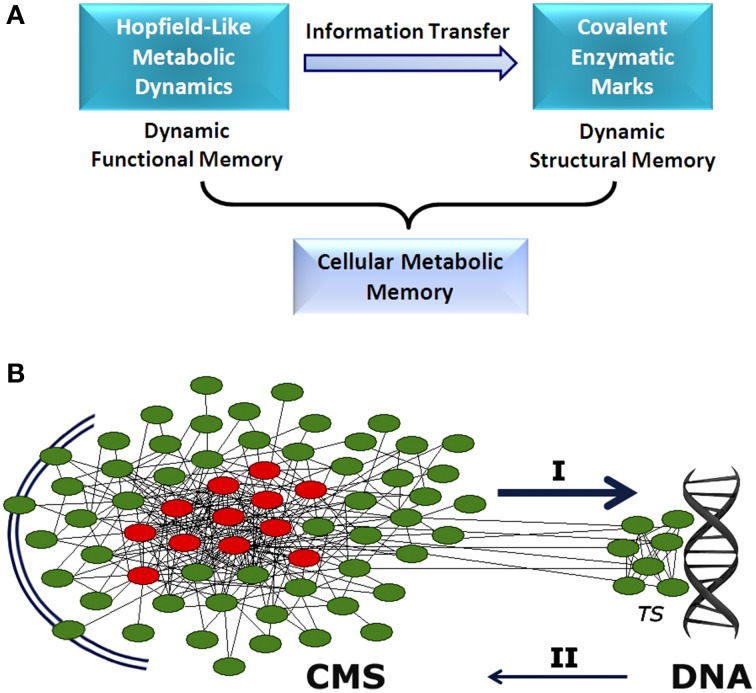
**Cellular metabolic memory. (A)** Hopfield-like attractor dynamics can emerge in metabolic networks. They have the capacity to store functional catalytic patterns which can be correctly recovered by specific input stimuli. These metabolic dynamics are stable and can be maintained as long-term memory. Specific information associated with these memories can be transferred to the enzymatic processes involved in covalent post-translational modulation. Thus, specific functional stored information can be reversibly embedded in the form of structural molecular marks. These two dynamical processes, storing of catalytic patterns in the form of metabolic attractors (functional processes) and stabilizing metabolic information as molecular marks (structural processes), represent the two stages of the dynamic memory of cellular metabolism. **(B)** Between the extracellular environment and DNA, there appears to exist a cellular metabolic structure (CMS) which behaves as a very complex decentralized information processing system with the capacity to store metabolic memory **(A)**. The CMS generates sets of biochemical instructions that make each enzymatic activity evolve with a particular and precise dynamic of change, allowing self-regulation and adaptation to the external medium. In addition, the CMS permanently sends a flow of molecular signals to DNA-associated metabolism, which shapes the complex transcriptional system. These molecular flows allow accurate regulation of gene expression, so that only specific polypeptides that are required for adaptive maintenance of the CMS are synthesized. Together, both informative systems (CMS and DNA) coordinate the physiological development of the cell. I: determinative molecular flux for the regulation of the transcriptional system. II: polypeptide reposition. *TS*: transcriptional system. The CMS is represented by a network. Red circles, metabolic core; green circles, on-off metabolic processes.

Different mechanisms are responsible for establishing functional metabolic integration. Studies of transfer entropy in DMNs have shown that the integration of specialized biochemical modules is mediated by effective connectivity processes which lead to the emergence of a systemic functional structure that encompasses the whole metabolic system. This global structure is characterized by a small set of different enzymatic processes which are always locked into active states (metabolic core), whereas the remaining catalytic reactions present on-off dynamics (see Figure 1A) (De la Fuente et al., 2011).

As early as in 1999, in an exhaustive analysis of several million different DMNs, the emergence of this functional structure at a global level was observed for the first time (De la Fuente et al., 1999). Afterwards, different studies implementing flux balance analysis of experimental data added new evidence of this systemic metabolic structure in the cell in which a small set of enzymatic reactions belonging to different metabolic processes remain active under all investigated growth conditions (metabolic core), and the remaining enzymatic reactions stay only intermittently active. The emergence of this global catalytic structure was verified for *E. coli, H. pylori*, and *S. cerevisiae* (Almaas et al., 2004, 2005). In these studies, the reactions present in the metabolic core were also characterized, and were found to be essential for biomass formation and for ensuring optimal metabolic performance. Likewise, the percentage of enzymatic reactions belonging to the core turned out to be small: 36.2% of all different reactions in *H. pylori*, 11.9% in *E. coli* and 2.8% in *S. cerevisiae*.

Similar results regarding the size of metabolic cores have been reported following extensive numerical analysis of DMNs, in which the percentages of metabolic subsystems pertaining to the core ranged from 44.6 to 0.7%. Nevertheless, their distribution was found to be skewed toward the lower percentages, since 71% of the studied networks showed metabolic core sizes ranging between 7.6 and 0.7% of all metabolic subsystems, with a mean size of 2% (De la Fuente et al., 2009).

In addition to the emergence of a global metabolic structure (De la Fuente et al., 2008), metabolic analysis by means of flux balance analysis and other studies of effective connectivity in DMNs have shown that two main dynamic mechanisms are involved in systemic metabolic self-regulation: *flux plasticity* and *structural plasticity* (Almaas et al., 2004, 2005; De la Fuente et al., 2011). Flux plasticity represents changes in the intensity of the substrate fluxes, whereas structural plasticity results in persistent changes in the dynamic structure conformed by the catalytic processes that present *on-off* and *on* changing states.

Another key player involved in functional metabolic integration is energy. Living cells require a permanent generation of energy flow to sustain the functionality of their complex metabolic structures, which integrate a large ensemble of modular networks. Practically all bioenergetic processes are coupled with each other via adenosine nucleotides, which are consumed or regenerated by different enzymatic reactions. Moreover, adenosine nucleotides also act as an allosteric control of numerous regulatory enzymes allowing changes in ATP, ADP, and AMP levels to regulate the functional activity of those metabolic subsystems that exhibit this allosteric mechanism. At a systemic level, the temporal dynamics of the concentrations of adenosine nucleotides seem to be determined by the adenylate energy system which represents the synthesis of ATP coupled to energy-consumption processes through a complex network of enzymatic reactions which interconvert ATP, ADP, and AMP (De la Fuente et al., 2014). Experimental studies have shown that adenosine nucleotide dynamics present complex transitions over time. Thus, the use of nanobiosensors has permitted real-time-resolved measurements of intracellular ATP concentrations in intact cells. These experiments revealed the rhythmic behavior of the concentration of ATP and complex quasi steady-state dynamics with large variations over time but, importantly, the concentration of ATP was never found to be constant (Ozalp et al., 2010; Ytting et al., 2012).

The concentration levels of ATP, ADP, and AMP roughly reflect the energetic status of the cell, and a specific systemic ratio between them was proposed by Atkinson as the adenylate energy charge (AEC) (Atkinson and Walton, 1967) which is a scalar index ranging between 0 and 1. When the total adenine nucleotide pool is in form of AMP, the energy charge is zero, and the system is completely discharged (zero concentrations of ATP and ADP). With only ADP, the energy charge is 0.5. If the whole adenine nucleotide pool is in form of ATP, the AEC is 1. Under physiological growth conditions, organisms seem to maintain their AEC within narrow values (between 0.7 and 0.95), despite extremely large fluctuations in adenine nucleotide concentrations. This systemic physiological phenomenon constitutes a key property of the functional integration of metabolic processes in the cell (De la Fuente et al., 2014). The principal biological significance of maintained AEC levels appears to be the necessity of having the adenylate pool highly phosphorylated, keeping the rate of adenylate energy production similar to that of adenylate energy expenditure (De la Fuente et al., 2014). In-depth experimental studies have shown that the physiological AEC ratio is practically preserved in all unicellular organisms, both eukaryotes and prokaryotes. Besides, this ratio is also strictly maintained during all metabolic transformations that occur during the cell cycle, thereby appearing to be a manifestation of the integrative processes of systemic metabolism (De la Fuente et al., 2014).

Functional specialization and integration are not exclusive; rather, they are two dynamic complementary principles, and the interplay between them seems to be an essential element for the functional collective architecture of systemic metabolism in the cell.

## Metabolic networks and information properties

### Experimental findings of biomolecular information processing in unicellular organisms

A large number of experimental studies have demonstrated that the processing and storing of some kind of biomolecular information is a fundamental requirement for cells to be able to exhibit complex systemic behavior.

One of the most studied examples of complex systemic behavior in a single cell is *Physarum polycephalum*. This unicellular species, belonging to the *Amoebozoa* phylum, is an important branch of eukaryote evolution, characterized by a large cytoplasm with many nuclei that remain suspended in a single contiguous protoplasmic volume called plasmodium. The body of the plasmodium contains a complex dynamic network of tubular structures by means of which nutrients and biomolecular elements circulate in an effective manner (Shirakawa and Gunji, 2007).

The movement of the plasmodium is termed shuttle streaming, characterized by the rhythmic back-and-forth flow of biomolecular substances. The dynamically reconfiguring network of tubular structures can redirect the flow of protoplasm toward the plasmatic membrane, causing the movement of a mass of flowing pseudopods, and as a result, the organism can crawl over the ground at a speed of approximately 1–5 cm/h (Kessler, 1982). The complex protoplasm exhibits rich spatio-temporal oscillatory behavior, and rhythmically synchronized streams with intracellular oscillatory patterns such as oscillations in ATP concentration, the plasmagel/plasmasol exchange rhythm and oscillations in the cytoplasmic free calcium level (Ueda et al., 1986).

Over the last several years, a number of studies of *Physarum polycephalum* have shown that this cell is able to store information, learn and recall past events. For example:

- This unicellular organism has the ability to find the minimum-length solution between two points in a maze; this feature is considered to require cellular information processing (Nakagaki et al., 2000, 2001); it has also been verified that the shortest path problem in the maze requires a mathematically rigorous solution (Miyaji and Ohnishi, 2008).
- *Physarum* constructs appropriate networks for maximizing nutrient uptake, achieving better network configurations than those based on the shortest connections of Steiner's minimum tree (Nakagaki et al., 2004a,b).
- In particular, high quality solutions for the 8-city traveling salesman problem (TSP), which is known to be NP-hard and hence computationally difficult, have been found by using *Physarum Polycephalum* (Aono et al., 2011a,b; Zhu et al., 2011).
- Other direct experimental studies found evidence that this kind of amoeba can memorize sequences of periodic environmental changes and recall past events during adaptation to different stimuli (it can even anticipate a previously applied 1 h cold-dry pattern) (Saigusa et al., 2008).
- Despite being a single multinucleate cell, the plasmodium can be used to control autonomous robots (Tsuda et al., 2007; Gough et al., 2009).
- This cell is able to form an optimized network closely approaching the purposely designed Tokyo railway system (Tero et al., 2010).
- Furthermore, it has also been verified that the plasmodium can solve complex multiobjective foraging problems (Dussutour et al., 2010; Bonner, 2010; Latty and Beekman, 2011).
- Self-organization, self-optimization and resolution of complex optimization problems by means of adaptive network development have also been reported in *Physarum Polycephalum* (Marwan, 2010).

All of these experimental results point to the need for an intrinsic memory storage mechanism (Marwan, 2010) and a “*primitive intelligence*” (Nakagaki et al., 2000; Nakagaki, 2001; Nakagaki and Guy, 2008; Saigusa et al., 2008; Pershin et al., 2009).

Many other experimental studies have shown that other unicellular organisms also exhibit sophisticated behavior including information storage. A pioneer scientist in revealing such behavior was Jennings, who showed many years ago that a single cell such as Paramecium can exhibit a primitive kind of learning and memory (Jennings, 1905). Neutrophils also exhibit a rudimentary memory system by which they are able to “recall” past directions (Albrecht and Petty, 1998). *Dictyostelium* and *Polysphondylium amoebae* seem to have a rudimentary memory and they can show long directional persistence (~10 min), being able to remember the last direction in which they turned (Li et al., 2008). Even individual neurons seem to display short-term memory, and permanent information can be stored when nerve cells in the brain reorganize and strengthen their connections with one another (Sidiropoulou et al., 2009).

At a molecular level, information processing (Ausländer et al., 2012; Daniel et al., 2013) has been observed in numerous processes, such as the reversible phosphorylation of proteins (Thomson and Gunawardena, 2009); microtubule dynamics (Faber et al., 2006); enzymatic processes (Baron et al., 2006; Katz and Privman, 2010); redox regulation (Dwivedi and Kemp, 2012); transcription (Mooney et al., 1998); genetic regulatory networks (Qi et al., 2013); input signal transduction (Roper, 2007); biochemical networks (Bowsher, 2011); NF-kappaB dynamics (Tay et al., 2010); intracellular signaling reactions (Kamimura and Kobayashi, 2012; Purvis and Lahav, 2013); metabolic switches (Ramakrishnan and Bhalla, 2008); the chemotaxis pathway (Shimizu et al., 2010); network motifs (Alon, 2007), and other cellular processes (Ben-Jacob, 2009).

### Quantitative studies for molecular information processing in self-organized enzymatic sets and in dissipative metabolic networks

Metabolic patterns may have information which can be captured by transfer entropy which allows the quantification of biomolecular information flows in bits (Schreiber, 2000). In 2012, the molecular informative properties of a single self-organized multienzymatic set (a metabolic subsystem) were analyzed *in silico* by using transfer entropy for the first time (De la Fuente and Cortés, 2012). According to this analysis, the information patterns contained in the different substrate fluxes are continually integrated and transformed by the set of enzymes that form part of the metabolic subsystem. Transfer entropy analysis also showed that in the integrative catalytic processes, a dynamic functional structure for molecular information processing emerges, characterized by changes in informative flows that reflect the modulation of the enzymatic kinetic activities. The level of functional influence in terms of causal interaction between the enzymes is not always the same, but varies depending on information fluxes and the particular dynamic regime of the catalytic system. The multienzymatic set shapes a functional dynamic structure, which is able to permanently send biomolecular information between its different enzymatic parts, in such a way that the activity of each enzyme could be considered an information event.

As a result of the overall catalytic process, enzymatic activities are functionally coordinated, and the metabolic subsystem operates as an information processing system, which at each moment redefines sets of biochemical instructions that make each enzyme evolve in a particular and precise catalytic pattern. In terms of information theory, the metabolic subsystem performs three simultaneous functions: signal reception, signal integration, and the generation of new molecular information. In light of this research, the self-organized enzymatic sets (metabolic subsystems) seem to represent the most basic units for information processing in cells (De la Fuente and Cortés, 2012).

On the other hand, when considering the activity of an interrelated set of different metabolic subsystems, a highly parallel, super-complex structure of molecular information processing emerges in the network. This was evidenced in a numerical study of dissipative metabolic networks in which the biomolecular information flows were quantified in bits between the metabolic subsystems by using transfer entropy (De la Fuente et al., 2011). Specifically, the results showed that the network as a whole integrates and transforms the molecular information coming from both the external environment and the individually assembled metabolic subsystems. The basic units of information processing (each self-organized multienzymatic set) work in parallel and coupled with each other, and as a consequence of these collective processes, functional modules, metabolic switches, highly dynamical metabolic synapses and an information processing super-system emerge in the network.

In addition to the topological network structure, characterized by the specific location of each multienzymatic set, molecular substrate fluxes and regulatory signals (mainly allosteric and covalent modulations), a functional network structure of effective connectivity emerges which is dynamic and characterized by having significant variations of biomolecular information flows between the metabolic subsystems. The dissipative network behaves as a very complex decentralized information processing super-system which generates molecular information flows between the self-organized enzymatic sets, and forces them to be functionally interlocked, i.e., each catalytic set is conditioned to cooperate with others at a precise and specific activity regime in concordance with the activity system as a whole. As a result of the overall functional process, the network defines sets of biochemical instructions at each moment that make every metabolic subsystem evolve with a particular and precise dynamic change of catalytic patterns. Quantitative analysis also revealed that the functional network is able to self-regulate against external perturbations by means of this information processing super-system (De la Fuente et al., 2011).

#### Dynamic metabolic memories

Memory is a fundamental requirement for efficient information processing. Recently, catalytic network dynamics have been numerically analyzed using statistical mechanics tools. In particular, an equivalent Hopfield network has been obtained using a Boltzmann machine, showing that enzymatic activities are governed by systemic attractors with the capacity to store functional metabolic patterns (De la Fuente et al., 2013). This stored molecular information can be correctly recovered from specific input stimuli (Table 3).

**Table 3:** **Metabolic dynamics and functional memory**.

**Fact**	**Result obtained**	**Consequences**
First modeling approach to metabolic networks by using Hopfield nets (De la Fuente et al., 2013)	I. Metabolic network dynamics evolve toward stable states (which are local minima of a Lyapunov function) II. Metabolic dynamics are governed by Hopfield-like attractors III. The metabolic system exhibits “pattern-completion” dynamics (i.e., when the network dynamics start within the basin of attraction, the network converges to one of the originally stored patterns)	I. Metabolic information patterns can be stored in the connectivity of the enzymatic network II. Stored catalytic patterns can be correctly recovered from specific input stimuli (i.e., associative memory)

In short, the main conclusions of the statistical mechanics analysis were the following:

- The catalytic dynamics of the dissipative metabolic network are dependent on the Lyapunov function i.e., the energy function of the biochemical system, which is a fundamental element in the regulation of enzymatic activities. Starting in any initial state following an external stimulus, and due to collective enzymatic dynamics, the metabolic network evolves in order to reduce the energy function, which in the absence of noise will decrease in a monotone way until achieving a final system state that is a local minimum of the Lyapunov function. When the biochemical system state reaches a local minimum, it becomes a (locally) stable state for the enzymatic network. The dissipative metabolic network has multiple stable states, each of which corresponds to a specific global enzymatic pattern of the biochemical system.
- Enzymatic activities of the metabolic network are governed by systemic attractors, which are locally stable states in which all enzymatic dynamics are stabilized. The minima of the Lyapunov function corresponds to these emergent systemic attractors, which regulate the metabolic patterns of the subsystems and determine the enzymatic network to operate as an individual, completely integrated system. The energy function, on which the dynamics of the metabolic network depend, has a complex landscape with multiple attractors (local minima).
- The emerging systemic attractors correspond to Hopfield-like attractors in which metabolic information patterns can be stored (Hopfield, 1982). The biochemical information contained in the attractors behaves as a functional metabolic memory i.e., enzymatic activity patterns stored as stable states, which regulate both the permanent catalytic changes in the internal medium, as well as the metabolic responses originated to properly integrate the perturbations coming from the external environment. When an external stimulus pattern is presented to the system, the network states are driven by the intrinsic enzymatic dynamics toward a determined systemic attractor which corresponds to a set of memorized biochemical patterns. The formation of the attractor landscape is achieved by metabolic synaptic modifications, i.e., the functional and molecular connections between enzymatic sets, essentially through substrate fluxes, covalent modulations (post-translational modulations) and allosteric regulations. These connectivity patterns can be oscillatory or steady states and comprises an infinite number of distinct activity behaviors. Each biochemical synapse is involved in the storage of the metabolic memory, and metabolic synaptic plasticity seems to be the basis of the memories stored in the metabolic network.
- A key attribute of the analyzed systemic attractors is the presence of “associative memory.” When the metabolic network is stimulated by an input pattern, enzymatic activities converge to the stored pattern, which most closely resembles the input, i.e., the attractors have associative memory. Metabolic network attractors seem to store functional enzymatic patterns which can be correctly recovered from specific input patterns. The results explicitly show that there are indeed memory attractors and a capacity to retrieve information patterns (from the weights) which are local minima of the catalytic activity in the network. Thus, if the initial condition of the network dynamics starts within the basin of attraction of such a pattern, the dynamics will converge to the originally stored pattern. As it is well known, this class of “pattern-completion” dynamics has been addressed in Hopfield network studies for years, and it is generally named “associative memory” (Amit, 1989).

#### The mean field approximation

In metabolic memory analysis, a mean field assumption was computed to describe the equivalent-Hopfield network depicting metabolic dynamics (De la Fuente et al., 2013). The results indicated that information can be stored and eventually retrieved by the local minima of the metabolic network energy; this is what researchers in neural network studies refer to as “having associative memory” (Amit, 1989). Notice that the original dissipative metabolic network can have much more complicated energy landscapes than the ones approached using the mean field solution. For more details about information encoding and retrieving (associative memory) and the capacity for pattern storage of a metabolic network see Supplementary Material S1.

### Post-translational modifications and metabolic memories

The dynamics of metabolic synaptic plasticity governed by Hopfield-type attractors are the fundamental element essential for storing functional metabolic memories in enzymatic networks. However, from a bioenergetic point of view, Hopfield-like metabolic memory is expensive since it requires permanent activity to maintain network functionality, spending substantial amounts of energy mainly in the form of ATP.

Recent investigations have shown that certain enzymes responsible for post-translational modulation can act on multimeric proteins which undergo different modification states that may have slow relaxation dynamics under specific conditions. The activity of such post-translational enzymes depends crucially on the temporal history of the molecular information they receive, and this information can be stored as a slow relaxation process in which slowness enables long-term maintenance of an embedded state (Hatakeyama and Kaneko, 2014). This property permits an efficient information transfer from the Hopfield-like metabolic dynamics to the enzymes involved in covalent post-translational modulation, so that functional memory can be embedded in multiple stable molecular marks.

Molecular information transfer from the functional metabolic memories to the covalent marks requires much less energy than the maintenance of emergent attractor dynamics in metabolic networks. Thus, the post-translational modification of proteins seems to be an essential mechanism for the maintenance of Hopfield-like memories in a structurally and energetically efficient way.

Post-translational modification (PTM) is a highly dynamic, essential enzymatic process which modifies protein functionality, reversibly transforming its structure by adding biochemical marks. Consequently, a single protein subjected to PTM may show a broad range of molecular functions and provoke profound physiological effects in cells (Cohen, 2000; Impens et al., 2014). In fact, PTM is a metabolic mechanism present in all cell types, which can affect the protein structures involved in most cellular processes, e.g., the regulation of the microtubule cytoskeleton (Janke and Bulinski, 2011); signal integration (Deribe et al., 2010); chromatin regulation (Bannister and Kouzarides, 2011); modifications of RNA silencing factors (Heo and Kim, 2009); modulation of mitochondrial membrane activities (Distler et al., 2009); regulation of different metabolic processes in the nucleus (Gloire and Piette, 2009); changes of cytochrome activity (Aguiar et al., 2005); regulation of the mitochondrial translation machinery (Koc and Koc, 2012) as well as other modulations of metabolic processes (Running, 2014). There are different types of PTMs, e.g., phosphorylation, methylation, glycosylation, acetylation, ubiquitination, SUMOylation, etc., and the number and abundance of these modifications is constantly being updated (Khoury et al., 2011).

Protein phosphorylation is one of the most extensively studied PTMs, and the phosphorylation-dephosphorylation mechanism is assumed to take place on a fast time scale in comparison to protein half-lives. Different sites on the same protein can be consecutively phosphorylated-dephosphorylated. These events are not distributed along the whole protein structure but are instead constrained to sites of high accessibility and structural flexibility (Gnad et al., 2007), which allows molecular information encoding, by adding or removing molecular marks (Thomson and Gunawardena, 2009).

While bare DNA encodes 2 bits of information per nucleotide, even in a single protein reversible chemical modifications occur at multiple sites, allowing the encoding of a potentially large amount of information. A protein with *n* phosphorylated sites has an exponential number (2∧ *n*) of phospho-forms; the number of phosphorylated sites on a protein shows a significant increase from prokaryotes (with *n* = 7 sites) to eukaryotes, with examples having *n* = 150 sites, implying that a protein can encode a large amount of molecular information as a function of a varying number of covalent phospho-marks (Thomson and Gunawardena, 2009). The reversible structural changes in regulatory networks of proteins through PTM allow molecular information encoding and complex information processing, which can originate flexibility and adaptive metabolic responses in the cell (Sims and Reinberg, 2008; Sunyer et al., 2008; Thomson and Gunawardena, 2009).

Attractor network dynamics followed by structural modifications of proteins would represent the two stages of the dynamic memory of cellular metabolism. In accordance with both processes, metabolic memories are derived from continuous changes in the functionality of the network dynamic (due to metabolic synaptic plasticity) linked to structural molecular modifications (covalent marks).

### Examples of post-translational modifications and molecular information storage in the cell

The *Escherichia coli* chemotaxis network represents one of the best studied examples of a biochemical dynamic system with the capacity to store functional information using molecular information processing linked to post-translational modification dynamics (Tu et al., 2008; Greenfield et al., 2009; Li and Stock, 2009; Shimizu et al., 2010; Stock and Zhang, 2013). Bacterial cell swimmers integrate environmental information accurately in order to make proper decisions for their own survival, in such a way that they can move toward favorable sites and away from unfavorable environments by changing their swimming patterns. As a result of the chemotaxis system, the signal transduction process translates information into appropriate motor responses and the bacterial cells traverse gradients of chemical attractants, displaying an efficient directional sensing and movement by performing temporal comparisons of ligand concentrations. Thus, the cell detects extracellular chemical gradients and senses very small fractional changes, even at nanomolar concentrations (Sourjik and Berg, 2002, 2004).

Since the pioneering studies by Julius Adler in the 1960's (Adler, 1966; Adler and Dahl, 1967; Adler and Templeton, 1967), the biochemical mechanisms that underlie sensory-motor regulation in *E. coli* have been extensively investigated and their principal characteristics have been detailed (Baker et al., 2006; Hazelbauer et al., 2008). Membrane receptors responsible for signal transduction assemble into large clusters of interacting proteins, shaping a complex modular network (Greenfield et al., 2009; Shimizu et al., 2010; Hamadeh et al., 2011) with one main feedback loop (Wadhams and Armitage, 2004; Emonet and Cluzel, 2008). The network consists of several thousand alpha-helical transmembrane protein fibers that interact with one another forming a cortical structure below the cytoplasmic membrane. The fiber ends that pass through the cytoplasmic membrane interact with a complex layer of sensory receptor domains.

*E. coli* sensory–motor functionality is regulated by two reversible protein modification processes: phosphorylation and carboxyl methylation. The phosphorylation processes provide a direct connection between sensory receptor complexes and motor responses. The transmembrane protein fibers shape a four-helix bundle with at least eight potentially anionic glutamate side chains that can be either exposed as a negative charge or neutralized by methylation. Each fiber can potentially be in any one of 2∧ 8 different states of modification, and the increments or decrements in attractant concentration produce behavioral responses that lead to changes in methylation dynamics (Stock and Zhang, 2013).

The dynamic changes in chemotaxis network functionality provoke specific molecular information processing and, as a consequence, structural molecular modifications in the form of methylation. They are carried out in such a way that the cell can record the recent chemical past by using this reversible methylation process in determined glutamic acid residues (Li and Stock, 2009). The dynamic methylation-demethylation patterns linked to information processing serve as a structural and functional memory allowing the detection of attractant gradients, by comparing current concentrations to those encountered in the past. The carboxyl methylation mechanism stores information concerning environmental conditions that the bacterium has experienced, and these dynamic patterns of glutamyl modifications act as a structural dynamic memory which allows cells to respond efficiently to continual changes in attractant concentrations (Greenfield et al., 2009; Li and Stock, 2009; Stock and Zhang, 2013).

The *E. coli* chemotaxis network exhibits a rich variety of behavior as well as dynamic properties such as robustness (Alon et al., 1999; von Dassow et al., 2000; Kollmann et al., 2005), signal amplification (Tu, 2013), molecular information processing (Shimizu et al., 2010), fast response, and ultra-sensitive adaptation (Bray et al., 1998; Wadhams and Armitage, 2004; Sourjik and Berg, 2004; Kollmann et al., 2005). The essential components of the *E. coli* chemotaxis system are highly conserved among all motile prokaryotes (Wuichet et al., 2007). However, many species have chemotaxis networks that are much more complex than that of *E. coli* (Hamadeh et al., 2011).

Although the chemistry of signal transduction in prokaryotic and eukaryotic cells differs substantially, there are numerous fundamental similarities (Stock et al., 2002). As in prokaryotes, signal transduction in eukaryotic cells generally involves phosphotransfer mechanisms and other post-translational modifications that couple metabolic energy to information processing (Li and Stock, 2009).

In particular, the biochemical systems of molecular information processing linked to post-translational modification dynamics are highly evolved in neuronal cells. Neurons are connected through synapses that undergo long-lasting activity dependent alterations in their transmission efficacy, permitting learning and response to experience. One of the principal means of controlling synaptic transmission is regulation of neurotransmitter receptors, especially the AMPA-type glutamate receptors which represent the principal excitatory neurotransmitter receptors in neurons. The number of AMPA receptors at the synapse is extremely dynamic and strictly regulated through the addition or removal of receptors resulting in long-term synaptic potentiation or depression (LTP or LTD, respectively) (Anggono and Huganir, 2012). These synaptic plasticity events are thought to underlie information storage. In particular, reversible post-translational modifications of AMPA receptors, including phosphorylation (Wang et al., 2005), palmitoylation (Hayashi et al., 2005), ubiquitination (Schwarz et al., 2010), SUMOylation (Luo et al., 2013), N-glycosylation (Tucholski et al., 2014) and O-GlcNAcylation (Taylor et al., 2014) are important regulatory mechanisms of synaptic AMPA receptor expression and function.

By controlling the expression and function of AMPA receptors, these multiple forms of post-translational modification can vigorously regulate plasticity events and many important functions linked to molecular information processing, including learning and memory (Takeuchi et al., 2014). While prevailing models of long-lasting information storage identify mRNA translation as essential for learning and memory in neural cells, there is evidence that memory storage at the PTM level can occur in the absence of protein synthesis (Routtenberg and Rekart, 2005; Routtenberg, 2008).

### Long-term and short-term memory

Since the beginning of the neuronal network modeling of associative memory, the connectivity matrix in the Hopfield network was assumed to result from a long-term memory learning process, occurring over a much slower time scale than neuronal dynamics (Willshaw et al., 1969, 1970; Amari, 1975; Hopfield, 1982; Amit, 1989; Hertz et al., 1991). Therefore, it is well accepted that the attractors emerging in neuronal dynamics described by Hopfield networks are the result of a long-term memory process.

Besides, extensive physiological recordings of neuronal processes have revealed some type of memory structure. Among others, there are at least two commonly used indicators for this kind of memory: the Hurst exponent (Das et al., 2003; De la Fuente et al., 2006) and the presence of long range correlations in plasticity dynamics for measured synaptic weight, for instance, long tails in the synaptic distribution of weights (Barbour et al., 2007). Several phenomenological models have shown that this persistence in the dynamics of weights underlying short-term memory might eventually be translated into a stable long-term memory after memory consolidation (Fusi et al., 2005; Mongillo et al., 2008; Itskov et al., 2011; Romani et al., 2013), but the precise mechanisms making this transition possible are not yet well understood.

Long-term correlations (mimicking short-term memory in neuronal systems) have also been analyzed in different metabolic processes not belonging to neuronal cell-type. One of the most studied is the calcium-activated potassium channels which has been observed in Leydig cells (Varanda et al., 2000; Bandeira et al., 2008), kidney Vero cells (Kazachenko et al., 2007), and human bronchial epithelial cells (Wawrzkiewicz et al., 2012). Other biochemical processes also present long-term correlation as for example, the intracellular transport pathway of *Chlamydomonas reinhardtii* (Ludington et al., 2013), the NADPH series of mouse liver cells (Ramanujan et al., 2006), and the mitochondrial membrane potential of cardiomyocytes (Aon et al., 2008). Moreover, different biochemical numerical studies on enzymatic pathways and dissipative metabolic networks also show long-term correlation (De la Fuente et al., 1998a,b, 1999). These studies support the thesis that, at the molecular level, metabolic memories exhibit both long-term and short-term memory, but this issue requires further research.

## The cellular metabolic structure—DNA system

### Elements of the cellular metabolic structure

Metabolism can be considered to be the largest known *source* of complexity in nature. Cellular enzymatic processes shape a highly structured, dynamic super-system with different levels of organization, which have a large number of adaptive molecular mechanisms acquired throughout biological evolution. On the way to structural and functional complexity, metabolic evolution has given rise to a large diversity of biochemical organizational forms, ranging from sophisticated bacterial metabolism, through the complex enzymatic networks belonging to protists, to the extraordinary structures and processes derived from multicellular eukaryotic organization, such as embryogenesis or the neural networks present in higher mammals. The millions of living metabolic species represent the inexhaustible source of complexity developed by the dynamic forces that structure the metabolic systems.

Enzymes are fundamental molecules for metabolic life. At a basic level, they can shape dissipatively structured functional associations (metabolic subsystems), which seem to constitute elemental catalytic units of the cell reactor. A rich variety of dynamic catalytic patterns emerges in each metabolic subsystem (including oscillatory and quasi-steady states), which correspond to distinct activity regimes (see Section Enzymatic Self-Organization). Catalytic changes allow metabolic synapses, i.e., the structural and functional dynamic connections between enzymatic sets. As a result of flexible activity regimes, flux plasticity and structural plasticity also appear in the cell (see Section Functional Organization of Enzymes in Metabolic Networks). At a superior level, enzymatic processes are organized into distinct modular metabolic networks, which work with relative independence of the global system. In turn, the whole metabolism seems to be functionally integrated, generating systemic responses from the concerted actions of modular networks, shaping a complex dynamic super-system, the Cellular Metabolic Structure (CMS). Therefore, the two principles that link the main modes of metabolic connectivity in the cell are functional segregation and functional systemic integration (see Section Functional Organization of Enzymes in Metabolic Networks).

The CMS is an integrated catalytic entity that acts as an information processing system capable of storing functional biochemical patterns. Metabolic synaptic dynamics governed by Hopfield-type attractors and structural covalent modifications represent the two stages of the dynamic memory of cellular metabolism. Plasticity seems to be the key essential property for the emergence of metabolic memory (see Section Metabolic Networks and Information Properties). All these properties constitute some of the main elemental principles of the CMS, which allow the self-organization, self-regulation and adaptation of the cell to the external medium.

### Self-regulation of the cellular metabolic structure

Cells are open metabolic systems. In order to maintain self-organized biochemical functionality, the CMS needs a permanent exchange of energy and matter with the external medium. As a consequence, metabolic life would be impossible in the absence of a capacity to adequately respond to the large number of combinations of external variables to which it is exposed.

The CMS is a sophisticated, sensitive system, whose enzymatic processes change immediately in response to variations in environmental conditions (Almaas et al., 2004, 2005; Papp et al., 2004; Wang and Zhang, 2009). When external perturbations are small, the CMS changes accordingly, and slowly reverts back to the pre-stimulus dynamic by reorganizing and adjusting catalytic patterns and metabolic fluxes (Inoue and Kaneko, 2013). If external perturbations are more intense, e.g., the replacement of a major source of nutrients with another, the CMS computes the various possible ways to efficiently process the molecular substrates, turning off certain metabolic activities (even completely eliminating specific enzymatic sets), and adding new reactive processes and alternative routes. As a result, metabolism shows extensive reorganization in its functionality, resulting in a rewiring of global network fluxes (Storlien et al., 2004; Buescher et al., 2012; San Román et al., 2014; Zhang et al., 2014).

When the metabolic system experiences extreme external conditions, e.g. nutrient deprivation, the CMS generates very complex enzymatic reorganization involving the entire system. In general, the autophagy interaction network is activated in response to starvation (Behrends et al., 2010), and as a consequence, proteins, organelles and other cellular substructures are sequestered in autophagosomal vesicles to be later degraded. This is one of the major mechanisms by means of which a starving cell reallocates nutrients from unnecessary metabolic pathways to more-essential processes (Shintani and Klionsky, 2004). Under starvation conditions, the CMS computes the efficient rates of molecular turnover, modulates systemic enzymatic processes with extreme precision, and adjusts the number of different organelles to match available resources. For instance, the CMS profoundly regulates different mechanisms such as ribosome biosynthesis and degradation (Pestov and Shcherbik, 2012), nucleolar proteome dynamics (Andersen et al., 2005), protein turnover (Neher et al., 2003), lipid degradation (Holdsworth and Ratledge, 1988), peroxisomal number (Mijaljica et al., 2006), and global cellular energy (Boender et al., 2011), among others.

There are endless possible combinations of environmental variables (physical, chemical, nutritional and biological) to which the cell must give an adequate response. The CMS has the properties to self-adapt in response to this plethora of external variables, displaying an impressive capacity to regulate thousands of metabolic processes simultaneously, computing the appropriate regulatory responses at each moment through complex and specific catalytic dynamic patterns that must represent an appropriate response to each set of external perturbations. Furthermore, these self-regulations occur very quickly. For instance, the metabolic dynamics of *Escherichia coli* were studied in cultures in which the glucose concentration was constantly changing, following an up-and-down pattern. The authors found that the systemic energetic state of the cell (measured by the adenylate energy charge) self-adjusted as a whole within only 2 min of the external perturbations (Weber et al., 2005).

In addition, metabolism also exhibits complex and continuous cycles of molecular turnover, including that of the complete proteome, glycome, lipidome, metabolome, and transcriptome of the cell. More specifically, enzymes, the essential molecular actors for cell functionality, are continually synthesized and destroyed in a dynamic turnover process in which all proteins are broken down partially into peptides, or completely, into amino acids for later *de novo* regeneration (Doherty and Beynon, 2006; Doherty et al., 2009; Li, 2010). As a result of the global metabolic dynamic that encompasses reactive molecular transformations, including protein turnover, specific polypeptides must be suitably synthetized when required by physiological conditions. Therefore adequate molecular information flow from the CMS to DNA is essential for ensuring adequate polypeptide replacement.

### Nexus between the cellular metabolic structure and DNA

Just over 40 years ago, the Nobel Prize Laureate John Gurdon demonstrated the existence of a molecular flow from the cytoplasm into the nucleus involved in the differential expression of certain genes during early embryonic development (Gurdon, 1971). His experiments involved transplantation of the nucleus of a cell, which was carrying out a specific dynamic activity, into an enucleated cytoplasm whose metabolic activity was quite another. These studies were an extension of Briggs and King's work on transplanting nuclei from embryonic blastula cells (Briggs and King, 1959).

Gurdon reached a fundamental conclusion on the basis of his experiments: that some cytoplasmic molecules control the expression of certain genes (or their repression) in an independent way. In all hybrid cells, the transplanted nucleus did not continue its previous activity, but rather changed its function to conform to that of the host cytoplasmic metabolism to which it had been exposed. For instance: the mid-blastula nucleus synthesizes DNA but not RNA, and when it is implanted into the cytoplasm of a growing oocyte whose nucleus does not synthesize DNA, but rather RNA, the new hybrid cell stops synthesizing DNA and starts synthesizing RNA. If this same mid-blastula nucleus is injected into the cytoplasm of a mature oocyte which is not synthesizing any nucleic acid and whose chromosomes are placed in spindle phase, the implanted nucleus ends up synthesizing DNA and the chromosomes arrange in the spindle of cell division. When an adult neuron nucleus, which synthesizes RNA but rarely synthesizes DNA, is injected into the cytoplasm of an unfertilized but activated egg whose nucleus would normally synthesize DNA but not RNA, the transplanted nucleus stops RNA synthesis and begins DNA synthesis (Gurdon, 1971).

Another one of Gurdon's experimental series proved that certain cytoplasmic components select which genes will be activated and/or repressed at any given time and consequently, it was deduced that major changes in different kinds of gene activity are a response to molecular signals coming from cytoplasmic metabolism (Gurdon, 1971). Analogous experiments have since been conducted in protozoans, amphibians, plants and in different mammals such as cats, mice, monkeys, rabbits, horses, donkeys, pigs, goats, and cows; even a mouse was cloned using the nucleus of an olfactory neuron (Eggan et al., 2004). Of all these, the most popular example is Dolly, who was the result of an experiment in which a single nucleus from a differentiated mammary cell was successfully fused with an enucleated unfertilized egg from another adult sheep (Wilmut et al., 1997).

According to contemporary paradigms, DNA contains all the essential information for the maintenance and development of the cell, and therefore the mammary nucleus should be able to modify the metabolic activity of the cytoplasm into which it has been injected, so that the new hybrid organism should act as a mammary cell. But what happened in the Dolly experiment was precisely the opposite: the transferred nucleus underwent a radical transformation via the molecular information flux which originated from the host cytoplasmic metabolism. This information reprogrammed genetic expression in the implanted nucleus which then began to express transcription patterns which were completely different to those associated with the previous, non-transplanted conditions. Overall, these experiments demonstrated that this new program of molecular instructions comes, not from the nucleus, but rather from the metabolic structure of the host cytoplasm.

In the Dolly experiment, gene expression patterns were regulated by the cytoplasmic metabolic structure of the cell into which the mammary nucleus had been injected. The hybrid cell no longer behaved like a mammary cell, but rather, it started to divide according to a new molecular program, that is, the sequence of instructions to perform a specified biochemical task, executed by cytoplasmic metabolism. During the first three cell divisions, the new cell replicates its DNA without expressing any genes, and this is followed by successive cell divisions and progressive differentiation, first into the early embryo and subsequently into all of the cell types that characterize the animal. Cellular Metabolic Structure (involving all the metabolism in both the cytoplasm and nucleoplasm) is able to reprogram the gene expression of mammary DNA, and both the CMS and the DNA coordinate together the development of an entire organism.

Extensive experimental observations of molecular flow from cytoplasmic metabolism to the nucleus of eukaryotic cells, date back to as early as the 1960s (Zetterberg, 1966; Arms, 1968; Gurdon and Weir, 1969; Merriam, 1969) and continue to be reported (Cook et al., 2007; Wimmer et al., 2013; Tamura and Hara-Nishimura, 2014). Many different experimental approaches have shown that molecular flow from cytoplasmic metabolism to the nucleoplasm consists of ordinary components of cellular metabolism. Thousands of proteins, metabolites, cofactors and ions are transferred to the nucleoplasm with remarkable efficiency and specificity (Kuersten et al., 2001; Cook et al., 2007; Gerhäuser, 2012), and the different molecular combinations, each with precise concentrations, lead to the modulation of DNA-associated metabolism, which regulates gene activity and other processes.

Extensive experimental studies both in prokaryotes and eukaryotes have uncovered the same fundamental process: genes exhibiting transcriptional induction in response to metabolic changes, having their origins in self-adaptation to a wide range of external factors such as nutrient deprivation, chemical agents (hormones, pH, ionic strength, among others) and physical fluctuations, including temperature shock, drought, high-intensity light etc. (Causton et al., 2001; Gasch and Werner-Washburne, 2002; Kreps et al., 2002; Hazen et al., 2003; Liu et al., 2005; Deutscher et al., 2006; Stern et al., 2007; Swindell et al., 2007; Wilson and Nierhaus, 2007; Angers et al., 2010; Mirouze and Paszkowski, 2011). For instance, under glucose starvation, the stringent reduction of ATP turnover at the metabolic level was accompanied by a strong down-regulation of genes involved in protein synthesis (Boender et al., 2011).

The traffic from the CMS to DNA-associated metabolism ensures the intimate subordination of the transcriptional system to the needs of metabolic activity, at each moment. The transcriptional system seems to be one of the most complex dynamic structures of the cell. It is made up of different, highly coordinated metabolic networks which integrate a large variety of signals and process molecular information from both the DNA and the CMS. Unfortunately, most of the dynamic regulatory aspects of the metabolic transcriptional system are still poorly understood.

A huge variety of biochemical mechanisms participates in the control of gene expression, including those which involve topoisomerases, transcription factors, RNA polymerases, small RNAs, the translation system, the turnover rate of proteins, the dynamic levels of adenylate energy charge, etc. These metabolic processes are in turn modulated by complex molecular networks (Balázsi et al., 2005; Missiuro et al., 2009) such as, for instance, those associated with miRNAs (Dong et al., 2010; Gennarino et al., 2012; Schulz et al., 2013), transcription factor processes (Walhout, 2006; Neph et al., 2012), RNA polymerase complexes (Schubert, 2014), and mRNA dynamics (Maquat and Gong, 2009; Schott and Stoecklin, 2010; Vlasova-St Louis and Bohjanen, 2011) among others.

Chromosome packaging is another essential biochemical control process for gene expression, and is common to all living organisms. In eukaryotes, nucleosome-based DNA organization allows for the reversible access of different metabolic regulatory mechanisms in order to modulate specific DNA sequences. In fact, the tails of the four core histones are subject to a variety of enzyme-catalyzed, post-translational modifications (Turner, 2005). Networks of biochemical factors that functionally interact with the nucleosome, reversibly remodeling it, have also been reported (Burgio et al., 2008). Prokaryotic cells, despite exhibiting a very different DNA organization to that of eukaryotes, also present a protein collectivity referred to as nucleoid-associated proteins, which contributes to the compaction and regulation of the genome (Rimsky and Travers, 2011; Takeda et al., 2011).

In addition, gene expression can also be modulated through methylation-demethylation dynamics on DNA nucleotides, mostly at CpG sites to convert cytosine to 5-methylcytosine. The enzymatic systems for these covalent marks are also integrated in DNA-associated metabolism, and seem to be directly involved in maintaining dynamic metabolic memories in the transcriptional system.

In brief, it appears that both eukaryotes and prokaryotes have a common mechanism to coordinate the two information storage systems in the cell: the CMS and DNA (Figure 1B). The CMS integrates and processes stimuli coming from the environment, generating self-regulatory metabolic responses and the traffic of molecular information to DNA-associated metabolism. This information flux ensures the intimate integration and subordination of the transcriptional system to the rest of the metabolic activity of the cell. The molecular flow allows the appropriate orchestration of the whole transcriptional system and, as a consequence, specific parts of the genome are activated and deactivated at strategic times so that, in accordance with the requirements of adaptive maintenance of cellular metabolism at each moment, gene expression is accurately regulated and only specific polypeptides are synthesized. Other important implications of the CMS in the regulation of DNA-associated metabolism, such as the generation of new polypeptidic information by means of alternative splicing or directed mutations, are beyond the scope of the present work.

### Duality of cellular hereditary information

Many different experimental approaches have provided ample evidence that specific functional metabolic processes codified via covalent mark patterns (epigenetic changes), can be preserved when cells divide and thus can be transferred to the next generation, in a manner which is different of DNA-based inheritance, i.e., this intergenerational preservation is not encoded in the DNA sequence.

For instance, epigenetic changes in response to environmental impacts on cellular metabolism have been observed to be transmitted to the offspring in different kinds of cells (Pembrey et al., 2006; Gluckman et al., 2007; Nadeau, 2009; Jiminez-Chirallon et al., 2009; Carone et al., 2010; Grossniklaus, 2011). Alterations in available nutrients may also induce specific metabolic processes that seem to be transmitted to the next generation by means of covalent marks that do not involve changes in the DNA sequence (Kaati et al., 2007; Waterland et al., 2007). Moreover, experimental findings regarding cells under stress shed new light on the transgenerational inheritance of metabolic memory processes (Seong et al., 2011; Crews et al., 2012; Pecinka and Mittelsten Scheid, 2012). Likewise, persistent and stable transmission of epigenetic information across generations has been reported. For instance, in *Arabidopsis thaliana*, environmental stress induces genomic flexibility (increasing the hyper-recombination state), which persists in a stable way for up to four untreated generations (Molinier et al., 2006).

A stably inherited DNA methylation pattern has also been reported in a variant of *Linaria vulgaris* originally described more than 260 years ago by Linnaeus (1749). In this example, the fundamental symmetry of the flower was found to change from bilateral to radial as a result of extensive methylation of the *Lcyc* gene that controls the formation of dorsal petals (Cubas et al., 1999).

The complex reprogramming of metabolic information marks in primordial germ cells according to their parental origin is another fine example of heritable biochemical patterns which are not caused by changes in the DNA sequence (Seisenberger et al., 2012; Hanna and Kelsey, 2014). The transmission of epigenetic metabolic information across generations has also been observed in bacteria (Casadesús and Low, 2013; Gonzalez et al., 2014).

Overall, epigenetic processes seem to be the manifestation of cellular metabolic memory. The covalent modification of histones and the dynamics of methylation-demethylation of specific DNA nucleotides appear to represent the storage of specific functional biochemical memories in a structural way, and these marks can emerge as a consequence of Hopfield-type metabolic dynamics.

There is thus ample evidence that cells have two types of information storage system which are capable of transmitting specific information to the following generations, i.e., the CMS and DNA systems. The molecular information stored in both systems may well be sufficient to coordinate the physiological development of the entire cell.

## Concluding remarks

The observations and consequent reflections I present here concerning numerous cellular processes and different quantitative biomolecular approaches, both presumably interrelated, have led me to consider, without underestimating the enormous importance of DNA, that the elements responsible for the organization and regulation of fundamental biological processes are to be found in determined properties and characteristics of cellular metabolism.

As far as I have been able to ascertain, I find it probable that the essential attributes upon which cellular functionality lies have their basis in the as yet insufficiently understood *Cellular Metabolic Structure*. In this paper, I have tried, in a conceptual manner, to present the basic elements of a dualist framework concerning the information stored in the cell, according to which the dynamic metabolic memory and the genetic memory seem to be the manifestation of two systems of information storage in the cell. From this perspective, rather than a *genoteque*^1^ in evolution, the cell could be considered to be a singular metabolic entity, able to permanently self-organize and self-regulate, as well as to adapt to the environment.

The work presented herein is necessarily and predominantly an exercise of multidisciplinary integration, where concepts from different branches of biological knowledge—mainly Biochemistry, Physiology, Cell Biology and Molecular Biology—are addressed within the approach of Systems Biology. Moreover, during the development and completion of quantitative studies, it has been necessary to analyze many experimental and numerical data with different analytic methods coming from several disciplines such as differential calculus, statistical mechanics, discrete mathematics, computing techniques and artificial intelligence, as well as other physico-mathematical tools. However, in this manuscript, mathematical formulation has been omitted in favor of a more didactic character of the text. That being said, it is necessary to stress that, in my opinion, without mathematics—the language of nature—it would not be possible to precisely understand metabolic dynamics and the catalytic functionality that sustains them. Fortunately, today the necessary application of mathematics to all fields of human knowledge, biology in particular, is widely accepted. In the autobiographical recollections that Charles Darwin wrote in 1876 for his children, he expressed the importance of understanding “something of the great leading principles of mathematics, for men thus endowed seem to have an extra sense” (Darwin, 1905).

The novel dual framework of functional and structural information stored in the cell which I present here could open new perspectives on the comprehension of subjects of notable interest, as yet not well understood, such as cellular differentiation, to name but one. Thus, cells with a different *Cellular Metabolic Structure* and, therefore, different catalytic activity programs, but with the same genetic heritage, can express different genes and manifest different physiological behavior. Something along these lines could be said about the Theory of Evolution, in terms of how it was presented by the endearing Charles Darwin, and the possibility that cells themselves are able to self-adapt to the environment permanently through the dynamic properties of cellular metabolism and the dual character of cellular hereditary information.

Frankly, I have to admit that dealing with these overwhelmingly complex issues of systemic cell biology with the desired clarity and precision is an extraordinarily difficult undertaking. In addition, our current comprehension of dynamic biological phenomena in its most elemental aspects is still very incomplete and insufficient. It is truly extraordinary that in such a reduced space of merely a few microns, millions of biochemical processes form a metabolic entity that is sensitive, self-organized, self-regulated, able to process and store information, and perpetuate itself in time.

### Conflict of interest statement

The author declares that the research was conducted in the absence of any commercial or financial relationships that could be construed as a potential conflict of interest.

## Footnotes

^1^Literally, a box of genes.
