# Supplementary material for: Elements of the cellular metabolic structure
Source: Front Mol Biosci. 2015 Apr 28;2:16. doi: 10.3389/fmolb.2015.00016 (PMC4428431; doi:10.3389/fmolb.2015.00016)
Supplement: Supplementary file 1 [file Presentation1.PDF]

Supplementary Material S1

1. Encoding and retrieving information patterns: associative memory

The synaptic connectivity matrix in the Hopfield network [De la Fuente et al., 2013], was the result of a Hebbian learning rule (firing together, wiring together) for which "simultaneous activation of two enzymatic subsystems leads to pronounced increases in biochemical synaptic strength between both catalytic sets".

The stimuli sequences of vectors which are encoded into the learning rule correspond to an information pattern. In the simplest case, the synaptic weights do not change their strength anymore after learning. When connecting enzymatic subsystems with the weights matrix, the stable states of the entire network dynamics coincide with one of the stimuli which were stored in the learning rule. Thus, the name of the information pattern comes from the fact that the information existing in the weight-encoded vector can be retrieved after learning by applying to the network a stimulus which is close to the one used for pattern encoding. Therefore, pattern information, encoding and retrieval exist in the dissipative metabolic network, which converts the biomolecular information flows into new activity patterns, modifies the efficiency in the connection between the self-organized multienzymatic sets, and retains these modifications with stability. This enzymatic dynamic behavior is governed by Hopfield-type attractors, and therefore, the dissipative metabolic network behaves as an attractor metabolic network which has associative memory properties.

Neural networks with associative memory, addressing storage and recollection of encoded patterns in the learning rule, have been studied in theoretical neurobiology for more than 50 years, starting with the preliminary work done by Willshaw [Willshaw et al., 1969] and continuing in several studies [Kohonen1972; Sejnowski 1977] finally being highly popularized by Hopfield [Hopfield 1982]. In these pioneering theoretical studies, it was suggested that such associative memory could explain the operational mechanisms for memory in neural systems; more recently the theoretical predictions formulated several decades ago have been validated in experiments on Hippocampus [Wills et al., 2005], when the existence of Hopfield attractors in neural circuits underlying associative memory was shown.

2. On the maximum capacity for storing information patterns in the metabolic network

Another important feature of an associative Hopfield network is the maximum number of patterns that can be stored, i.e., the storage capacity. Studies of this issue on Hopfield networks have been widely analyzed and discussed [Hopfield 1982; Hertz et al., 1991; Amit 1992; Peretto 1992]. Under many different network assumptions and encoding rules, beyond specific model details, the storage capacity for *recollection memory*, requiring a full retrieval of the complete encoded memory, approximately scales with N (the number of nodes) [Hertz et al., 1991].

Far fewer studies exist on the storage capacity of Hopfield networks regarding *familiarity memory*, the possibility that the neural network can discriminate previously encoded patterns from new patterns which have not been encoded before [Bogacz and Brown 2003; Greve et al., 2010]. For this "yes or no" discrimination task, the storage capacity has a different nature in the scaling size; it scales with N∧2 versus the N scaling occurred for recollection [Bogacz and Brown 2003; Cortes et al., 2010; Greve et al., 2009]. Thus, the same Hopfield network can perform both recollection (full pattern retrieval) and familiarity (the discrimination between one memory which was previously encoded or a new one, never seen before), and very interestingly, the different memory nature has a very different storage capacity (N∧2 for familiarity vs N for recollection).

A rough estimation of the minimal magnitude order of the functional metabolic memories stored (recollection memory) in prokaryote cells, e.g., E. coli, will be above 100,000 (the familiarity metabolic memory would be much greater with N∧2), whereas the number of genes in this bacteria is around 4,500 [De la Fuente et al., 2013].

Eukaryotic cells are larger and more structured than prokaryotic cells, with larger genomes and essentially much more complex metabolic systems. An estimated minimum magnitude order for metabolic memories stored in these cells would be greater than several million (recollection memory), and familiarity memory would be much greater still [De la Fuente et al., 2013].

In addition to the genetic information, the metabolic networks of living cells seem to have a greater amount of biomolecular information in the form of functional metabolic memory stored in the connectivity pattern dynamics of the self-organized enzymatic sets.

The enzymatic activities seem to be governed by Hopfield-type attractors with capacity to store metabolic information patterns which can be correctly recovered by the specific input stimuli. As a consequence, the dissipative multienzymatic network has the capacity to learn, self-regulate and self-adapt to external conditions [De la Fuente et al., 2013].

References

Amit, D.J. (1992). *Modeling Brain Function: The World of Attractor Neural Networks.* New York: Cambridge University Press.

Bogacz, R., and Brown, M.W. (2003). Comparison of computational models of familiarity discrimination in the perirhinal cortex. *Hippocampus*. 13: 494-524.

Cortes, J.M., Greve, A., Barrett, A.B., and van Rossum, M.C.W. (2010). Dynamics and robustness of familiarity memory. *Neural. Comput*. 22:448-466. doi: 10.1162/neco.2009.12-08-921.

De la Fuente, I.M., Cortes, J.M., Pelta, D.A., and Veguillas, J. (2013). Attractor metabolic networks. *PLoS One*. 8(3):e58284. doi: 10.1371/journal.pone.0058284.

Greve, A., Davidson, D.I. and van Rossum, M.C.W. (2010). A single-trace dual-process model of episodic memory: a novel computational account of familiarity and recollection. *Hippocampus*. 20: 235-251. doi: 10.1002/hipo.20606.

Greve, A., Sterratt, D.C., Donaldson, D.I., Willshaw, D.J., and van Rossum, M.C.W. (2009). Optimal learning rules for familiarity detection. *Biol. Cybern*. 100: 11-19. doi: 10.1007/s00422-008-0275-4.

Hopfield, J.J. (1982). Neural networks and physical systems with emergent collective computational abilities. *Proc. Nat. Acad. Sci. USA*. 79(8):2554-2558. doi: 10.1073/pnas.79.8.2554.

Hertz, J., Krogh, A. and Palmer, R.G. (1991). *Introduction to the theory of neural computation*. Boston: Addison-Wesley Longman Publishing Co.

Kohonen, T. (1972). Correlation Matrix Memories. *IEEE Trans. Comput*. C-21:353-359. doi: 10.1109/TC.1972.5008975.

Peretto P (1992) An Introduction to the Modeling of Neural Networks. Cambridge University Press.

Sejnowski, T.J. (1977). Storing covariance with nonlinearly interacting neurons. *J Math. Biol*. 4(4):303-321 203-211. doi: 10.1007/BF00275079.

Wills, T.J., Lever, C., Cacucci, F., Burgess, N., and O'Keefe, J. (2005). Attractor dynamics in the hippocampal representation of the local environment. *Science*. 308: 873-876. doi: 10.1126/science.1108905.

Willshaw, D.J., Buneman, O.P., and Longuet-Higgins, H.C. (1969). Non-holographic associative memory. *Nature*. 222, 960-962. doi: 10.1038/222960a0.
